# Metabolomic Profiling of Native Yucatan Maizes (*Zea mays* L.): A Strategy for Harnessing Biodiversity

**DOI:** 10.1021/acs.jafc.5c09711

**Published:** 2026-01-06

**Authors:** Elia María Ku Pech, Emilio Piña Betancourt, Claudia Balderas, Javier Orlando Mijangos-Cortés, Enrique Sauri Duch, Sonia de Pascual-Teresa

**Affiliations:** † Departamento de Metabolismo y Nutrición, 231772 Instituto de Ciencia y Tecnología de Alimentos y Nutrición (ICTAN), Consejo Superior de Investigaciones Científicas (CSIC), Jose Antonio Novais 6, Madrid 28040, Spain; ‡ 16379 Centro de Investigación Científica de Yucatán, Chuburná de Hidalgo, 97205 Mérida, Yucatán, México; § 27743 Instituto Tecnológico de Mérida, Tecnológico Nacional de México, 97118 Mérida, Yucatán, México; ∥ 201430 Instituto de Química Orgánica General (IQOG), Consejo Superior de Investigaciones Científicas (CSIC), Juan de la Cierva 3, Madrid 28006, Spain

**Keywords:** anthocyanins, native maize, phenolic compounds, metabolomics, biodiversity, sustainable agriculture

## Abstract

Maize (*Zea mays* L.) is a globally significant crop. In the Yucatan Peninsula, native races such as Tuxpeo, Dzit Bacal, and Nal Tel exhibit unique genetic diversity and are rich in phenolic compounds. This study characterized the bioactive composition of these maizes, focusing on phenolic and antioxidant activity. From the 100 maize populations collected, 24 were selected based on intense coloration (13 purple (P) and 11 red (R)). Analyses included colorimetry, total soluble phenols (TSP), antioxidant capacity (FRAP and DPPH), and high-performance liquid chromatography with mass spectrometry (HPLC-QTOF-MS) for metabolomic profiling. Results revealed significant differences in phenolic content and antioxidant activity among populations, with the Chac chob 6 (R) population showing the highest TSP (1.45 mg g^–1^) and FRAP (3.17 mg g^–1^) values. Metabolomics identified 512 molecular entities with anthocyanins dominating in purple maize and other flavonoids in red maize. Multivariate analyses revealed distinct metabolic profiles among the populations.

## Graphical abstract

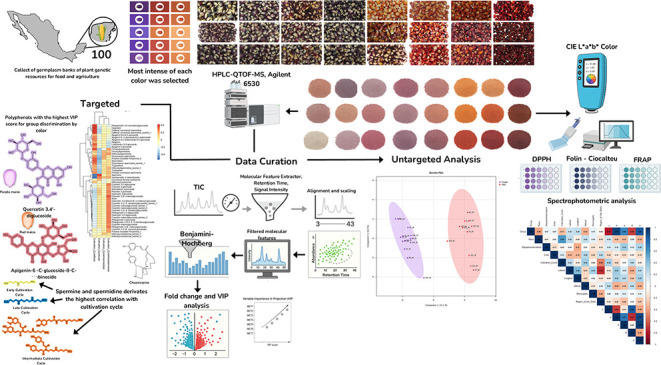

## 1 Introduction

Agriculture plays a fundamental role in achieving the sustainable development goals, particularly in terms of food security and hunger eradication.^1^ Among the most important crops worldwide, maize (*Zea mays* L.) stands out as the most widely produced cereal, with production continuously growing due to its versatility and adaptability.^2^ Maize can be cultivated in almost all agricultural regions under various production systems, and throughout most of the year, making it a cornerstone for global nutrition.^3^ Additionally, maize is a nutritionally complete food, providing complex carbohydrates, fiber, vitamins, minerals, proteins, fats, and significant amounts of bioactive compounds.^4^

Mexico is one of the largest producers of this cereal globally. Out of the 647 known varieties worldwide, Mexico is home to 59 native varieties which hold unparalleled historical, economic, and nutritional significance. Since ancient times, maize has been the foundation of Mesoamerican diets and its genetic represents an invaluable cultural and biological heritage.^5^ The Yucatan Peninsula is home to three native maize races—Tuxpeño, Dzit Bacal, and Nal Tel—whose interbreeding has resulted in a rich diversity of cobs, distinguished by their unique shapes, sizes, flavors, and colors, collectively known as local varieties.^6^ The flavors and colors of these varieties are closely linked to their chemical composition particularly the presence of phenolic compounds including flavonoids.^7^ These bioactive compounds not only contribute to the sensory characteristics of maize but also might offer significant health benefits. They act as free radical scavengers, preventing oxidative stress and reducing the risk of chronic diseases such as cardiovascular disorders, diabetes, and neurological disorders.^8^ Additionally, phenolic compounds have anti-inflammatory and antimicrobial properties, making them valuable in promoting overall health, for this reason, the regular consumption of polyphenol-rich foods, such as maize, is highly recommended for human health.^9^

Despite the nutritional and cultural importance of maize, there is a notable lack of scientific information about the composition and concentration of phenolic and related compounds in native maize varieties from the Yucatan Peninsula. This knowledge gap limits the sustainable use, conservation, and genetic improvement of these phytogenetic resources.^10^

Therefore, the objective of this study was to investigate and characterize the bioactive chemical composition of native maize cultivated in the Yucatán Peninsula using metabolomic profiling, for the first time as far as we know. This approach aimed to evaluate its nutritional quality, enhance the value of Yucatecan native maize, and contribute to a better understanding of its biochemical diversity.

## 2 Materials and Methods

### 2.1 Collection of Genetic Materials

The collection of genetic material was carried out from January to March 2020. The sampling strategy was based on the guidelines for germplasm banks of plant genetic resources for food and agriculture.^11^ The state of Yucatan was divided into three regions: the south, central, and east. Samples were collected in 43 communities across 18 municipalities in the state of Yucatan (Supporting Figure S1), resulting in a total of 100 native maize populations collected. They were stored in the Germplasm Laboratory (Germolab) at the Yucatan Center for Scientific Research (CICY). From the 100 populations, the 24 with the most intense coloration were selected, resulting in 13 purple (P) and 11 red (R) populations (Table 1). The color was verified by using color charts from the Royal Horticultural Society.

**Table 1:** Population Number, Variety, Color, Cultivation Cycle, Municipality, and Region of the State for the Studied Grains^a^

population number^d^	race/varie^b^	color^c^	cultivation cycle^d^	municipality	region of the state
006-R	Chac chob	A-Greyed purple	late	Tahdziú	South
007-P	Ek ju’ub	A-Greyed purple	late	Tahdziú	South
008-R	Chac chob	A-Greyed purple	late	Tahdziú	South
015-P	Ek ju’ub	A-Greyed purple	late	Peto	South
020-R	Nal tel	A-Greyed purple	early	Tixmehuac	South
021-P	Ek ju’ub	A-Greyed purple	early	Tixmehuac	South
042-P	Ek ju’ub	A-Violet blue	late	Valladolid	South
051-P	Ek ju’ub	A-Purple	late	Yaxcabá	Central
056-P	Ek ju’ub	A-Violet blue	late	Peto	South
060-P	Ek ju’ub	A-Violet blue	late	Peto	South
063-R	Chac chob	A-Greyed purple	late	Peto	South
064-P	Ek ju’ub	A-Violet blue	late	Peto	South
067-R	Nal xoy	B-Greyed orange	intermediate	Sotuta	Central
080-P	Ek ju’ub	A-Purple	late	Oxkutzcab	South
084-P	Ek ju’ub	A-Purple	late	Oxkutzcab	South
103-P	Ek ju’ub	A-Purple	late	Espita	South
105-R	Chac nal	B-Greyed orange	late	Uayma	South
107-R	Chac xim	A-Greyed purple	late	Temozón	South
108-P	Ek ju’ub	A-Greyed purple	late	Sotuta	Central
111-R	Xnuk nal	A-Greyed orange	late	Kaua	South
113-R	Dzit bacal	B-Greyed orange	intermediate	Peto	South
114-R	K’an pok	B-Greyed orange	late	Tizimin	South
115-R	Nal tel	A-Greyed purple	early	Peto	South
116-P	Nal tel	A-Purple	early	Peto	South

a Population number and coloration: R (Red) and P (Purple).

b Mayan nomenclature used by farmers during collection.

c Based on the classification levels of the color chart (Royal Horticultural Society, fifth ed.).

d Vegetative cycle indicated by farmers, where Early is less than 70 days, Intermediate is between 70 and 90 days, and Late is greater than 90 days.

### 2.2 Sample Pretreatment

The grain underwent a drying process at room temperature in the shade and under chemical disinfestation using aluminum phosphide tablets. Subsequently, the grain was grounded to obtain flour using a toothed disc mill and sieved to a particle size of less than 100 μm.

### 2.3 Selection of Intensity, Homogeneity, and CIE L a* b* color

A color chart (Royal Horticultural Society, fifth edition) was used to visually characterize the grain coloration based on intensity and homogeneity. The comparison was performed by using 10 randomly selected grains from the middle section of the cob. Each grain was matched to the closest color tone on the chart. Under natural light from a lamp, each maize grain was placed against the color chart for an accurate assessment. The dry sample was analyzed colorimetrically by using a Jf Lhabo model W*R*-10QC digital colorimeter. The color values L a* and b* are dimensionless and represent luminosity, redness-greenness, and yellowness-blueness, respectively. The samples were analyzed in their ground form. Measurements were taken in triplicate. Chromaticity (Cr) and hue (°Hue) were calculated.

### 2.4 Extraction of Bioactive Compounds

Extractions were performed using 50 mg of maize sample with 1 mL of high-performance liquid chromatography (HPLC)-grade methanol and ultrapure water (50:50, with 0.1% formic acid). The mixture was vortexed at 1250 rpm for 30 s (Eppendorf MixMate) and sonicated for 15 min, followed by centrifugation at 10,000 rpm and 4 °C for 10 min (Eppendorf Model 5415 R). The supernatant was recovered, and the residue was re-extracted twice by adding 0.5 mL of MeOH: water (50:50, with 0.1% formic acid) each time. Finally, the three supernatants were combined. Extractions for each maize sample were performed in triplicate.

### 2.5 Characterization of Bioactive Compounds

#### 2.5.1 Total Soluble Phenol Content

The previously obtained extract was used to determine the total soluble phenolic content following the method described by Singleton and Rossi (1965). A volume of 10 μL of the extract was mixed with 150 μL of the Folin-Ciocalteu working solution. After 3 min, 50 μL of a saturated sodium bicarbonate solution (75 mg mL^–1^) was added. A gallic acid standard curve (50–600 μg mL^–1^) was used for quantification. Ultrapure water served as the blank. The plate was incubated for 2 h at room temperature in the dark. Absorbance was measured at 725 nm using a PowerWave HT visible spectrophotometer (BioTek, Madrid, Spain). Results were expressed as milligrams of gallic acid equivalents (GAE) per gram of sample. Analyses were performed in triplicate for each sample.

#### 2.5.2 Ferric Reducing Antioxidant Power

The FRAP method, as described by Benzie and Strain (1996), was followed. The FRAP reagent was prepared using a 10:1:1 ratio of acetate buffer, 2,4,6-Tri­(2-pyridyl)-s-triazine (TPTZ), and FeCl_3_. A volume of 10 μL of each sample (in triplicate) was mixed with 290 μL of the buffer mixture. The incubation time was 15 min at 37 °C. Absorbance was measured at 593 nm using a PowerWave HT plate reader (BioTek, Madrid, Spain). A trolox solution was prepared by dissolving 25 mg of the compound in 100 mL (25 mL of 96% ethanol and 75 mL of distilled water). Analyses were performed in triplicate for each sample. Results were calculated based on standard curves and expressed as micrograms of Trolox equivalents per gram of sample (mg g^–1^).

#### 2.5.3 DPPH Radical Scavenging Assay

To determine the antioxidant capacity of the maize samples, we performed the 2,2-diphenyl-1-picrylhydrazyl (DPPH) assay was performed. A stock solution of DPPH (1 mmol L^–1^) was prepared by dissolving 39.4 mg of DPPH in 100 mL of methanol, stored at −18 °C, and protected from light. A diluted solution of 100 μmol L^–1^ was prepared from the stock solution. In each plate well, 10 μL of the sample was mixed with 290 μL of the diluted DPPH solution. The plate was incubated for 1 h in the dark with agitation using a MiniMix Labnet Inc. shaker. Absorbance was measured at 517 nm using a PowerWave HT plate reader (BioTek, Madrid, Spain).

### 2.6 Identification and Quantification of Phenolic Compounds Using HPLC-QTOF-MS

To determine the content and identification of compounds in maize grains, such as flavonols, phenolic acids, and anthocyanins, high-performance liquid chromatography coupled to mass spectrometry (HPLC-MS; Agilent Technologies, Madrid, Spain) was used. The chromatograph, equipped with a quaternary pump (G1311A), was coupled to a diode array detector (Agilent G1315D) and an LC/MS Agilent 6530 Accurate-Mass Q-TOF instrument with electrospray ionization (ESI) and Jet Stream technology (Agilent Technologies).

Compounds were separated using a Phenomenex Luna C18 column (3 μm, 4.6 mm × 150 mm) maintained at 25 °C. Water-formic acid (99.9/0.1) and acetonitrile-formic acid (99.9/0.1) were used as solvents A and B, respectively. The injection flow rate was 0.5 mL min^–1^, with the following gradient for solvent B: 10% at time 0, 30% at 30 min, 35% at 35 min, 45% at 40 min, followed by the equilibration of the column for 15 min (see an example of total ion chromatogram (TIC) in Supporting Figure S2). The injection volume was 5 μL. ESI parameters were as follows: drying gas temperature of 325 °C; drying gas flow rate of 8 L min^–1^; nebulizer pressure of 45 psi; sheath gas temperature of 300 °C; sheath gas flow rate of 11 L min^–1^; capillary voltage of 4000 V; and fragmentor voltage of 120 V. ESI was operated in positive mode, and data were collected in an extended dynamic range of 100–1700 *m*/*z*.

Spectral signals were obtained at 280, 360, and 520 nm. MassHunter Qualitative Analysis B.07.00 software (Agilent Technologies, Madrid, Spain) was used to compare the mass spectra and retention times with the corresponding standards. For compounds without available standards, identification was based on the chemical formula derived from accurate ion mass measurements and a comparison with bibliographic references related to maize. Additionally, public databases such as HMDB, FooDB, and PubChem were accessed to compare the exact masses and fragmentation patterns, when available. For quantification purposes, a calibration curve was prepared using standards (concentration range: 3.125–100 μg mL^–1^) to quantify the compounds, expressed as micrograms per gram of sample for epicatechin and quercetin (flavonols) and cyanidin-3-glucoside (anthocyanins).

### 2.7 Statistical and Metabolomic Analysis

For the spectrophotometric analysis, the results were expressed as the means of three replicates ± standard deviation. A one-way analysis of variance was performed, and the comparison of means used the minimum significative difference with *p* ≤ 0.05, RStudio version 1.2.5109 (RStudio Team, 2019) was used.

After data acquisition, noise and adducts were evaluated using the molecular feature extraction algorithm to collect all ions proceeding from the same compound, grouping them into the same signal, which was identified as a molecular entity; all molecular entities were characterized by a unique retention time, mass, and abundance. Parameters for filtering abducts included profile/centroid spectra ≥1000 counts for + H, + Na, + K, + NH4 with an absolute height ≥5000 counts. Data were subjected to batch recursive analysis, alignment, and sample grouping. All data underwent extraction with a peak to peak height of 1000 counts and charge state of two; the alignment using a retention time and mass used a time gap of 0.5% ± 0.15 min and 10 ppm ± 2.0 mDa.

Prior to data analysis, scaling and normalization were performed to ensure that metabolite concentration differences were interpretable and comparable. Data transformation (Log10 normalization) and scaling (autoscaling) were applied. Autoscaling adjusts each value by subtracting the mean value and dividing it by the variable’s standard deviation. This method assigns equal importance to all variables, which is particularly useful when variables differ greatly in magnitude.

The untargeted metabolomic analysis profiles were performed by MassHunter Qualitative Analysis (B.07.00), MassHunter Profinder (B.10.00), Agilent Mass Profiler Professional (B.15.1), Unscrambler (version 3.10), MetaboAnalyst (6.0), and the results were compared with the Human Metabolome Database (2022) and FooDB (Version 1.0).

For targeted metabolomic profiling, univariate analysis (UVA) was conducted on the 48 molecular identities. To assess the magnitude of expression changes between conditions, a Fold Change analysis was performed. To determine significant differences between experimental groups, a *t* test was applied. For visualizing and selecting significant differences between conditions, highlighting variables with the largest changes and statistical significance, a volcano plot was constructed with *p* < 0.05 and Fold Change (FC) > 2. For multivariate analysis (MVA), Principal Component Analysis (PCA) was performed to observe sample clustering. Pareto scaling was applied to the data prior to PCA (this step was also executed in Unscrambler for visualization purposes). To identify variables most influential in group separation, Partial Least Squares Discriminant Analysis (PLS-DA) was conducted. Variable Importance in Projection (VIP) scores were obtained for each metabolite in the PLS-DA model. Compounds were considered significant if VIP > 1. Finally, all compounds were visualized using a heatmap, enabling global data interpretation. Red color indicates increased metabolite levels in a sample, while blue represents decreased levels. For the univariate analysis the normality was determined by Shapiro-Wilk Test and to reduce the false positive we applied the Benjamini-Hochberg test.

## 3 Results and Discussion

### 3.1 Homogeneity and CIE L*a* b Color

The results obtained in the color analysis (Figure 1) show that maize flour presented a dark tone, as evidenced by the L values. All of the red maize flours had a significant intense color. The population with the highest *a was 113 that shows a red-orange color. Population 60 was the most colored. Some populations classified as gray orange in color were found to be the ones with the highest chroma value (≥26). In the purple maize the chroma values ranged from 11.00 to 16.00, while in the red maize the values were in the range from 17.73 to 39.00 as maximum observed.

**Figure 1:**
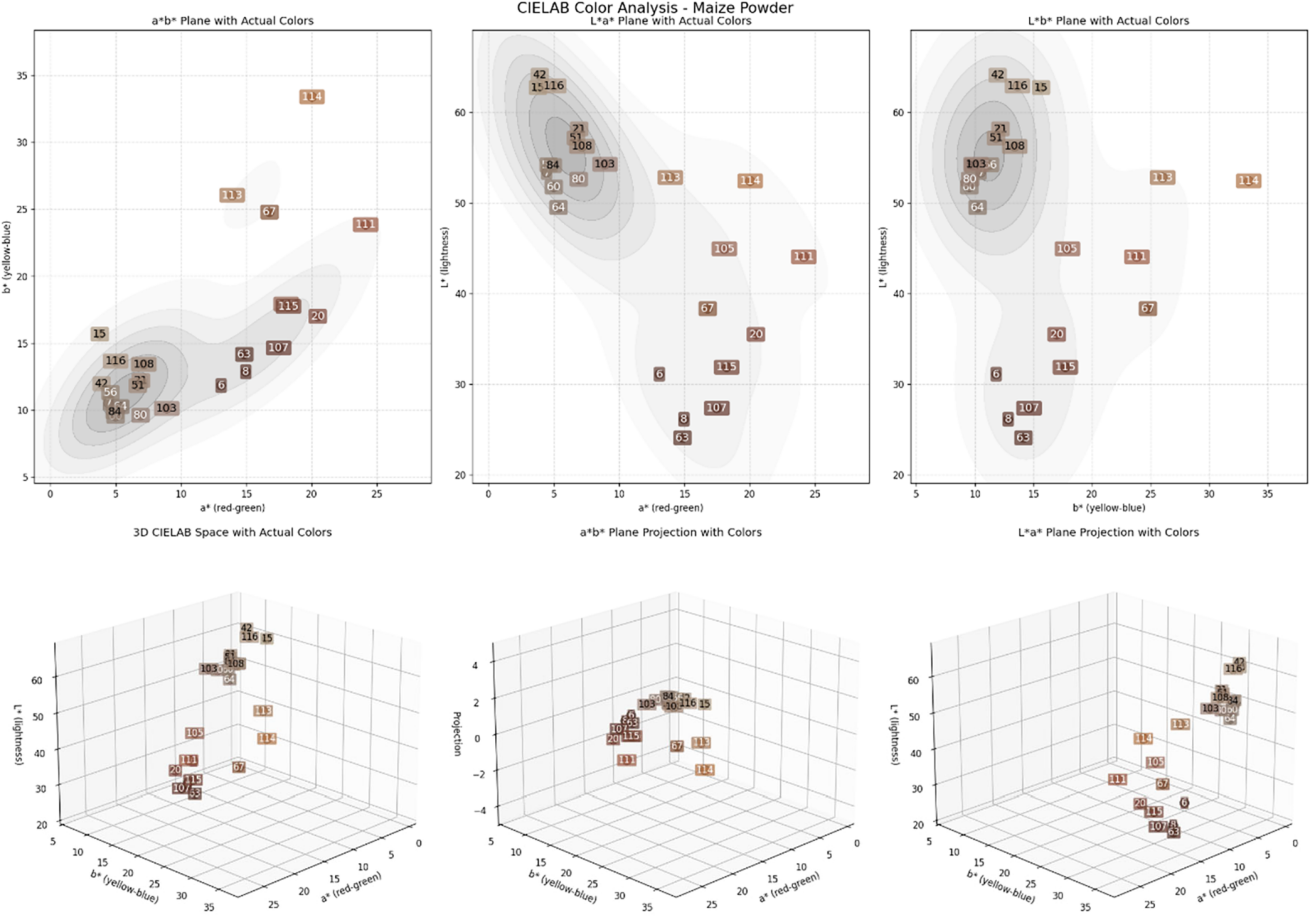
CIE L*a*b color distribution of the maize flour from different populations. L means Luminocity, tendency to white color, *a is green-red, tendency to red color, and *b is blue-yellow tendency to yellow color.

### 3.2 Total Polyphenols Content and Antioxidant Activity

Significant differences (*p* ≤ 0.05) were observed in total soluble polyphenol content (TSP), antioxidant activity (FRAP) and DPPH* radical scavenging activity among the 25 native maize populations analyzed (Table 2). Six (R) recorded the highest TSP value (1.45 ± 0.02 mg EAG g^–1^), while 21 (P) showed the lowest TSP value (0.58 ± 0.00 mg EAG g^–1^). In the case of FRAP, 6 (R) stood out with the highest value (3.17 ± 0.04 mg g^–1^) in contrast with 113 (R) which presented the lowest value (0.89 ± 0.02 mg g^–1^). For antiradical activity measured by DPPH*, 6 (R) also stood out (3.16 ± 0.14 mg g^–1^), while populations 15 (P) and 21 (P) showed the lowest values (0.67 ± 0.05 and 0.54 ± 0.04 mg g^–1^), respectively.

**Table 2:** Total Soluble Phenols (TSP) Content and Antioxidant Capacity (FRAP and DPPH Radical Scavenging) in 25 Native Maize Populations^a^

pob	TPF (mg EAG g^–1^)	CV %	FRAP (mg g^–1^)	CV %	DPPH* (mg g^–1^)	CV %
6 (R)	1.45 ± 0.02^a^	1.27	3.17 ± 0.04^a^	1.40	3.16 ± 0.14^a^	4.53
7 (P)	1.01 ± 0.01^bcde^	1.21	1.95 ± 0.05^b^	2.61	1.48 ± 0.14^defghi^	9.59
8 (R)	0.97 ± 0.01^bcdefg^	1.37	1.52 ± 0.05^cdef^	3.50	0.73 ± 0.04^hi^	5.30
15 (P)	0.75 ± 0.00^hijk^	0.00	1.07 ± 0.06^ghi^	5.96	0.67 ± 0.05^i^	7.23
20 (R)	1.11 ± 0.06^b^	5.21	1.84 ± 0.04^bc^	2.33	0.96 ± 0.05^fghi^	5.39
21 (P)	0.58 ± 0.00^k^	0.00	0.92 ± 0.04^i^	4.88	0.54 ± 0.04^i^	8.20
42 (P)	0.88 ± 0.02^cdefgh^	2.21	1.43 ± 0.06^defgh^	4.46	0.74 ± 0.05^ghi^	6.22
51 (P)	0.67 ± 0.03^jk^	3.79	1.31 ± 0.06^efghi^	4.86	2.34 ± 0.17^abcdef^	7.37
56 (P)	0.82 ± 0.03^defghij^	3.21	1.25 ± 0.00^efghi^	0.00	1.47 ± 0.09^cdefgh^	6.39
60 (P)	0.73 ± 0.01^hijk^	1.33	1.18 ± 0.02^fghi^	1.35	1.14 ± 0.01^defghi^	1.01
63 (R)	1.06 ± 0.05^bc^	4.56	1.77 ± 0.10^bcd^	5.55	1.05 ± 0.07^fghi^	6.68
64 (P)	0.74 ± 0.02^ghij^	2.64	1.16 ± 0.03^fghi^	2.32	1.70 ± 0.09^efghi^	5.47
67 (R)	0.62 ± 0.01^ijk^	1.94	0.99 ± 0.04^i^	4.09	1.03 ± 0.03^fghi^	3.37
80 (P)	0.76 ± 0.02^fghij^	2.94	1.14 ± 0.01^efghi^	0.54	1.73 ± 0.14^efghi^	8.36
84 (P)	0.87 ± 0.06^cdefghi^	6.84	1.43 ± 0.08^defg^	5.83	0.86 ± 0.07^hi^	7.92
103 (P)	0.65 ± 0.06^jk^	9.75	1.13 ± 0.09^ghi^	8.04	2.60 ± 0.07^ab^	2.76
105 (R)	0.79 ± 0.03^efghij^	4.35	1.32 ± 0.08^efghi^	6.25	2.81 ± 0.11^abc^	3.74
107 (R)	0.93 ± 0.02^bcdef^	1.79	1.57 ± 0.11^cde^	6.72	2.46 ± 0.02^abcde^	0.93
108 (P)	0.75 ± 0.05^ghij^	6.78	1.20 ± 0.01^efghi^	0.51	1.64 ± 0.08^bcdefg^	4.79
111 (R)	0.76 ± 0.02^fghij^	2.44	1.06 ± 0.05^hi^	5.12	1.20 ± 0.04^efghi^	3.17
113 (R)	0.65 ± 0.01^jk^	1.59	0.89 ± 0.02^j^	1.91	1.94 ± 0.07^bcdefgh^	3.77
114 (R)	0.64 ± 0.02^jk^	2.99	1.00 ± 0.07^i^	6.93	1.89 ± 0.12^bcdefg^	6.59
115 (R)	0.99 ± 0.05^bcd^	4.99	1.79 ± 0.05^bcd^	2.74	2.77 ± 0.22^abcd^	7.86
116 (P)	0.95 ± 0.07^bcde^	7.44	1.50 ± 0.05^cdefg^	3.50	1.03 ± 0.08^ghi^	8.11

a Values are expressed as mean ± standard deviation (*n* = 3). Means within a column that do not share a common letter are significantly different (*p* ≤ 0.05).

These results are in agreement with previous studies in which the anthocyanin content of a given food or sample is associated with the purple color and a superior antioxidant activity. The presence of compounds with high antioxidant activity in the Yucatecan maize could reflect undocumented local adaptations.^12^

Populations 6 and 7 exceeded the levels previously reported in commercial maize varieties.^13^ This finding suggests that native Yucatán populations may serve as valuable dietary sources for combating oxidative stress.^14^ Furthermore, the observed correlation between CIE Lab color intensity and total phenolic content supports the idea that pigmentation can serve as a practical visual marker for selecting varieties with potential nutraceutical value.

### 3.3 Characterization of the Composition by HPLC-QTOF-MS

The initial untargeted analysis detected 1,861 molecular features, each representing a distinct metabolic feature and in all potentially representing a metabolic fingerprint from each maize. To refine the data set, sequential filters were applied: first, retention time (RT) constraints (3.2–43 min) reduced the features to 1,183; next, a relative standard deviation (RSD) filter (<20 ppm mass error) further narrowed the results to 1,123 high-confidence entities. Finally, manual curation excluded poorly defined or noisy peaks, yielding a refined set of 512 well-resolved molecular entities for subsequent statistical and metabolic profiling.

#### 3.3.1 Univariate Analysis

The univariate analysis of the Fold Change (Figure 2) identified significant changes in the relative concentration of cyanidin malonylglucoside (FC: 100.32; log2­(FC): 6.65), quercetin malonylglucoside (FC: 89.57; log2­(FC): 6.48), and cyanidin-3,5-diglucoside (FC: 44.21; log2­(FC): 5.47) in contrast with apigenin that showed a significant reduction (FC: 0.022; Log2­(FC): −5.45).

**Figure 2:**
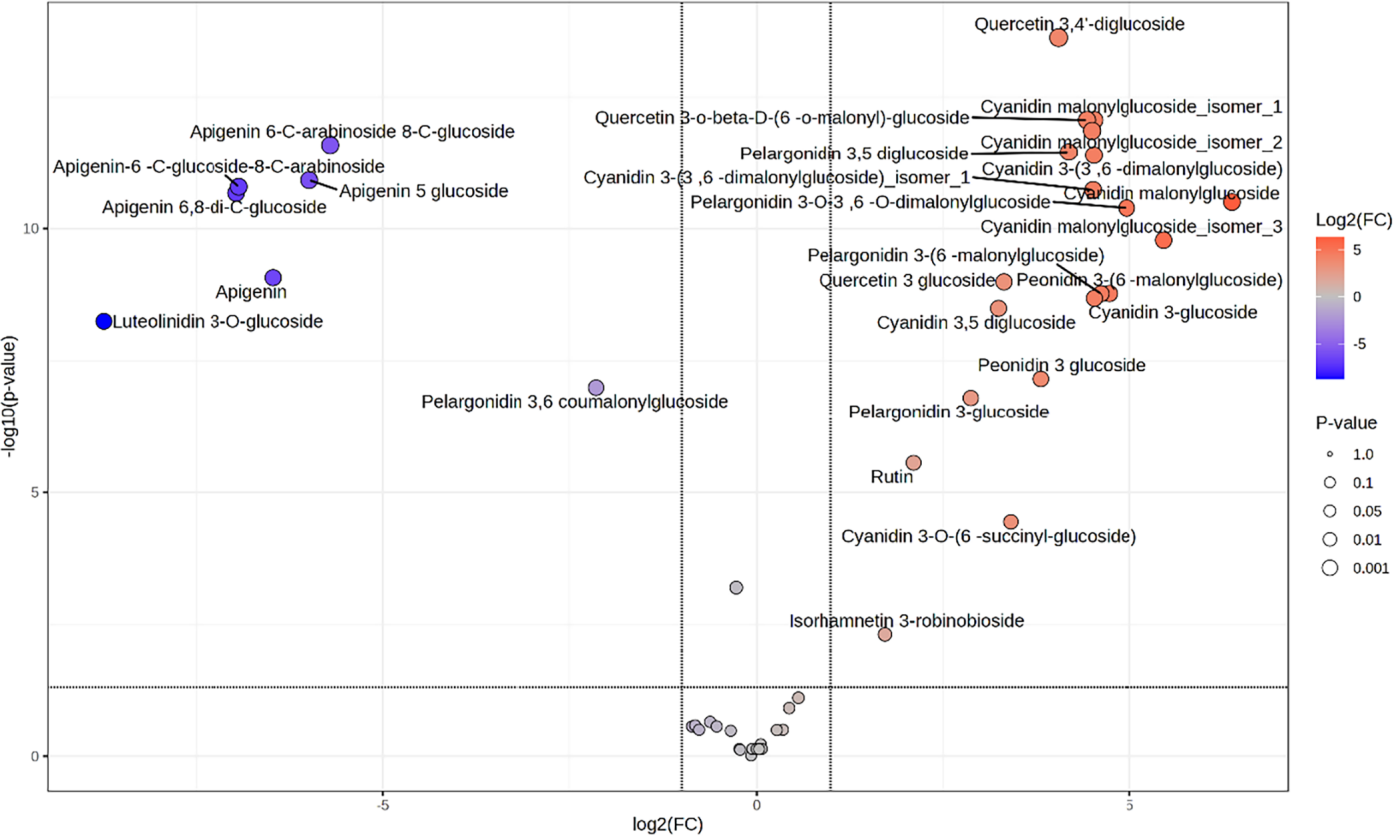
Important features selected by volcano plot with fold charge threshold (*x*) 2 and *t* tests threshold (*y*) 0.05. The red circles represent features above the threshold. Note that both fold charges and *p* values are log transformed. The further its position away from (0,0), the more significant.

The student′s *t* test confirmed the significant differences in the concentration of several metabolites between groups. The compounds with the most pronounced differences between groups were pelargonidin-3,5-diglucioside, cyanidin malonylglucoside and quercetin malonylglucoside. The precedent results show a significant difference in the metabolomic profile of red and purple maize populations with almost 300 features identified as statistically significant different. The purple maize was characterized by a high presence of anthocyanins, especially cyanidin-3-glucoside and peonidin-3-glucoside while the red maizes show more flavonols, including quercetin, quercetin-3-glucoside and kaempferol-3-glucoside.

The clear separation observed in the metabolomic profiles, as explained by the PCA analysis (53.4% variance), supports the presence of distinctive genetic biodiversity in the Yucatán Peninsula.^14^ This is particularly significant given the threats faced by native varieties, such as agricultural homogenization and climate change.^15^

#### 3.3.2 Clustering and MANOVA

Principal Component Analysis (PCA) revealed a clear separation of samples based on grain color, with the first component accounting for 52.1% of the variance and the second accounting for 25.8%. This clustering aligned closely with the metabolomic profiles of the maize samples. Additionally, Partial Least Squares Discriminant Analysis (PLS-DA) identified key metabolites contributing to the differentiation among groups (Figure 3).

**Figure 3:**
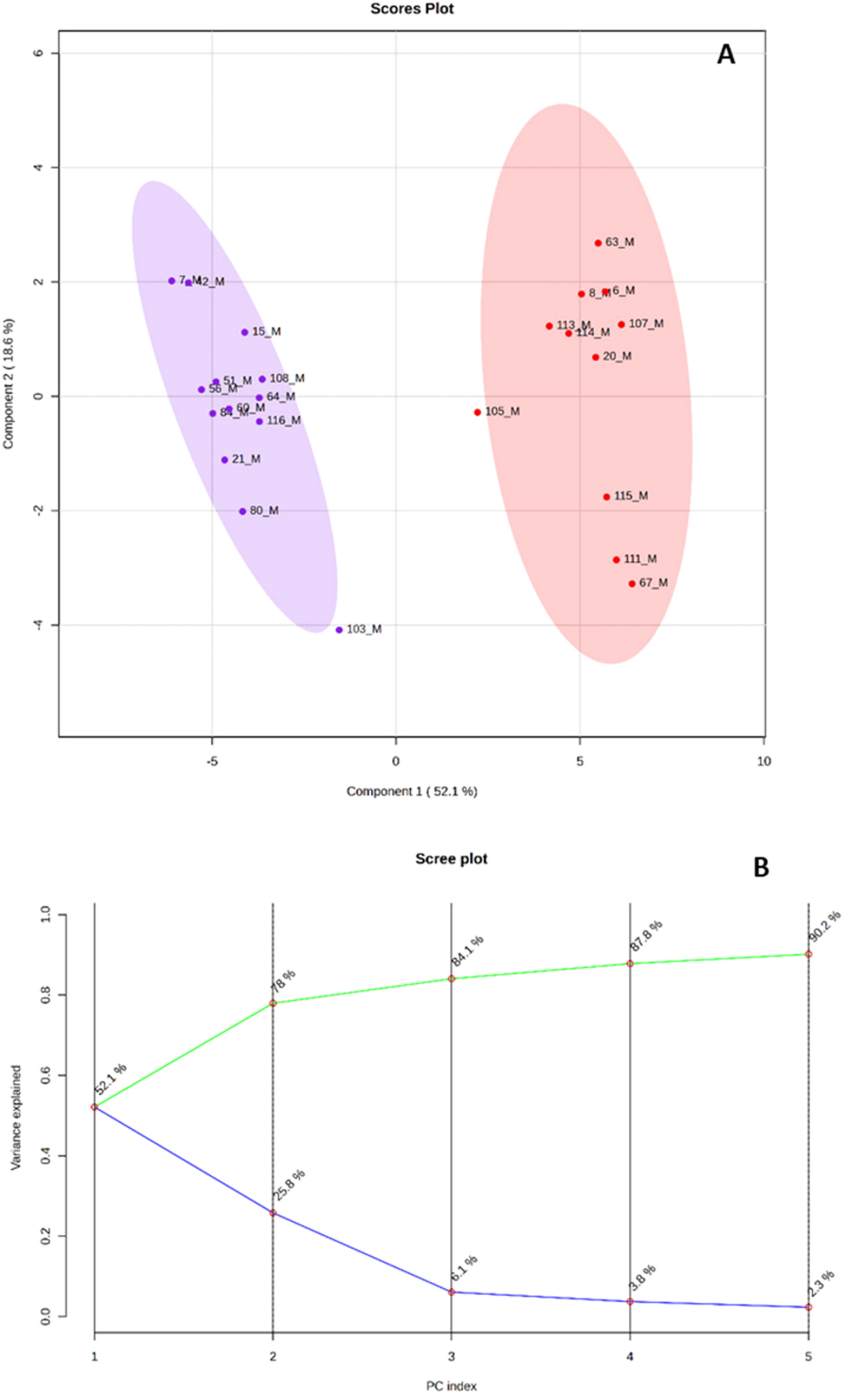
(A) Principal component analysis (PCA) of the metabolomic profile of red (red circle solid) and purple (blue circle solid) maize. Score plots (PCA) show the grouping of corn samples by color. The ellipses represent 95% confidence intervals. (B) Scree Plot Variance explained by each principal component (PCA) in the metabolomic analysis of native maize, showing the cumulative variance (71.8%).

The results of the metabolomic analysis evidence the dominant presence of anthocyanins in the purple maize and flavonols in red maize, suggesting a differential regulation of the phenylpropanoid biosynthetic routes of corn.^16^ The overproduction of cyanidin malonylglucoside is a common specificity in maize and can explain their high antioxidant capacity.^17^ In contrast, the reduction of apigenin in red purple maize suggest a metabolic imbalance toward the synthesis of flavonols instead of flavones, a phenomenon documented in maize varieties exposed to abiotic stress.^18^

The metabolomic heterogenicity in each color group shows up that similar varieties might have a unique chemical diversity.^19^ The populations with high concentrations of acylated compounds are candidates to develop varieties with thermostable antioxidants useful in processed foods, as it has been shown that acylation introduces stability in the molecules.

#### 3.3.3 Identification of Key Metabolites in Maize

The study identified 48 compounds (Table 3), classified into three major groups: anthocyanins and derivatives, flavonols and flavones, and amines, polyamines, and phenolic amides. Multiple isomers were detected, particularly among acylated anthocyanins and polyamine conjugates, reflecting the chemical diversity of the native maize populations.

**Table 3:** Identified Metabolites in Native Maize Populations: Retention Times, Masses, and Fragmentation Patterns

ID	identification	RT	MoMs	MS/MS+
1	spermine	2.09	202.2157	
2	spermidine	2.28	145.1579	
3	trigonelline	2.9	137.0477	
4	cyanidin-3,5-diglucoside	4.15	611.1612	611.1675, **449.1041**, 385.7888, **287.0524**, 73.9845
5	feruloylspermidine	4.2	321.2052	
6	pelargonidin-3,5-diglucoside	4.32	595.1663	595.1591, **433.1065**, **271.0630**, 156.8676
7	feruloyl putrescine	8.14	264.1474	265.1544, **177.0530**, 145.0274, 117.0290
8	tryptophan	8.27	204.0899	205.0970, 188.0672, 146.0603, 132.0780, 91.0520
9	cyanidin-3-glucoside	10.03	449.1084	449.1081, 288.0573, **287.0544**
10	luteolinidin-3-glucoside	10.35	433.1135	433.1130, **271.0574**, 243.0445 143.0283
11	pelargonidin-3-glucoside	12.07	432.3810	433.1130, **271.0578**
12	cyanidin malonylglucoside isomer I	12.78	535.1088	
13	peonidin-3-glucoside	13.13	463.1240	463.1352, 379.8255, **301.0716**, 222.8641
14	cyanidin malonylglucoside isomer II	13.91	535.1088	
15	chaenorpine	13.44	492.2733	493.2806,348.1293, 322.1597, 280.092, 265.0851, 214.1914, 169.1306
17	apigenin-6,8-di-C-glucoside	15.35	594.1585	595.1744, **577.1524**, **457.1115**, 427.0985, **337.0661**
18	cyanidin malonylglucoside	15.36	535.1088	535.1091, **449.1099**, **287.0533**, 218.9739
19	cyanidin-3-O-(6″-succinyl-glucoside)	15.64	549.1244	549.1254, 492.2113, **449.1055**, **287.0544**, 219.1837
20	caffeoyl coumaroyl spermidine	16.42	453.2277	454.2329, 308.1947, **292.2018**, 221.1241, **163.0378**, 147.0434
21	caffeoyl coumaroyl spermidine Isomer I	16.71	453.2277	
22	pelargonidin-3-(6″-malonylglucoside)	17.46	519.1139	520.331, 288.0611, 272.0549, 163.0761, 127.0364
23	cyanidin-3-(3″,6″-dimalonylglucoside)	18.07	621.1092	622.1561, **287.053**3, 279.1010, 193.0620
24	dicoumaroyl spermidine	18.12	437.2315	
25	peonidin-3-(6″-malonylglucoside)	18.24	549.1244	
26	apigenin-6-C-glucoside-8-C-arabinoside	18.25	564.1479	565.1522, **547.1446**, **529.1366**, **427.1017**, 409.0894, 391.0793
27	cyanidin-3-(3″,6″-dimalonylglucoside) isomer I	19.05	621.1092	
28	dicoumaroyl spermidine Isomer I	19.21	437.2315	
29	apigenin-6-C-arabinoside 8-C-glucoside	19.4	564.1479	565.1551, **547.1408**, **529.1328**, **427.0964**, 379.0805
30	diferuloylspermidine	19.5	497.2526	498.2595, 322.2114, 234.1117,248.1222, 177.0577, 146.0279
31	cyanidin malonylglucoside Isomer II	19.87	535.1088	
32	feruloyl-coumaroyl-spermidine	19.97	467.2420	468.2497, 292.2013, **234.1094**, 177.0534, **147.0427**,**119.0448**
33	diferuloylspermidine Isomer I	20.57	497.2526	498.2595, 322.2114, 234.1117,248.1222, 177.0577, 146.0279
34	pelargonidin-3,6-coumalonylglucoside	20.74	579.1503	579.1649, **433.1113**, 337.0672, **271.0572**, 85.0272
35	pelargonidin-3-O-3″,6″-O-dimalonylglucoside	21.5	605.1143	
36	rutin	22.37	610.1534	611.1605, **465.1721**, **303.0477**, 163.0282, 129.0521
37	apigenin 5-glucoside	22.39	432.1056	433.1131, 415.1017, 337.0700, 313.0700, 283.0580, 271.0589
38	quercetin-3,4′-diglucoside	23.11	626.1483	
39	quercetin-3-glucoside	23.95	464.0955	465.1022, 305.0527, **303.048**, **153.0166**, 124.0329
40	Isorhamnetin 3-robinobioside	26.25	624.1690	
41	quercetin-3-(6″-o-malonyl)-glucoside	26.33	550.0959	
42	kaempferol-3-glucoside	27.36	448.1006	
43	diferuloyl putrescine isomer I	31.9	441.2024	
44	diferuloyl putrescine isomer II	34.43	441.2024	
45	coumaroyl feruloyl putrescine	35.38	410.4700	411.1922, 265.1504, 235.1432, **177.0529**, **147.0429**, 119.0491
46	diferuloyl putrescine	36.19	441.2024	441.2014, **265.1510**, **177.0528**, 145.0264, 72.0770
47	quercetin	38.67	302.0427	303.0851, 285.1681, 242.0497, 177.0524, 153.0170, 136.5426
48	apigenin	43.37	270.0528	

The most abundant compounds were cyanidin, pelargonidin, and peonidin glycosides, including acylated forms, such as cyanidin malonylglucoside isomers and cyanidin-3-(3″,6″-dimalonylglucoside). Quercetin derivatives—quercetin-3-glucoside and quercetin malonylglucoside—along with apigenin conjugates like apigenin-6-C-glucoside-8-C-arabinoside were also prominent. Free apigenin levels were comparatively low, suggesting preferential glycosylation in these varieties.

Polyamines and phenolic amides were identified, with spermine and spermidine detected alongside their acylated derivatives, including feruloylspermidine, diferuloylspermidine, and caffeoyl coumaroyl spermidine isomers. Other bioactive compounds, such as tryptophan and trigonelline, were also detected.

A compound at *m*/*z* 493.2806 ([M + H]^+^) exhibited fragmentation patterns (Supporting Figure S3) with a main fragment at *m*/*z* 265.0851, 214.1914, and 169.1306. This compound was present in all maize samples analyzed in this and previous works by our research team.^20^ It did not absorb at any of the selected ultraviolet–visible (UV–vis) wavelengths (280, 360, and 520 nm) and under our conditions resisted fragmentation, and the main MSMS peak continued to be at *m*/*z* 493.2806. Finally, this peak at *m*/*z* 493.2806 ([M + H]^+^) was identified as the spermine alkaloid chaenorpine based on the previous work by Zhang and Peterson (2018) whom identified it based on NMR and Mass Spectrometry results and reported it as being within the primary bitter compounds in corn.^21^ Within this framework, polyphenols, together with polyamines, phenolic amides, and alkaloids, are likely to contribute to the unique sensorial and functional attributes characteristic of each maize population. This observation emphasizes the considerable yet underexplored chemical diversity of the Yucatecan native maize metabolomes.

#### 3.3.4 Targeted Analysis

The CIE L*a*b* colorimetric parameters showed significant correlations with chemical profile parameters that have previously been linked to bioactive compound concentrations. The strong association between the a* component (redness tendency) and red maize (*r* = 0.93) confirms that flavonoids such as apigenin contribute to reddish hues. The negative correlation between lightness (L*) and purple maize (*r* = −0.79) reflects the darkening effect of anthocyanins (Figure 4). These relationships suggest that color parameters could serve as indirect indicators of metabolic profiles and agronomic traits.^22^

**Figure 4:**
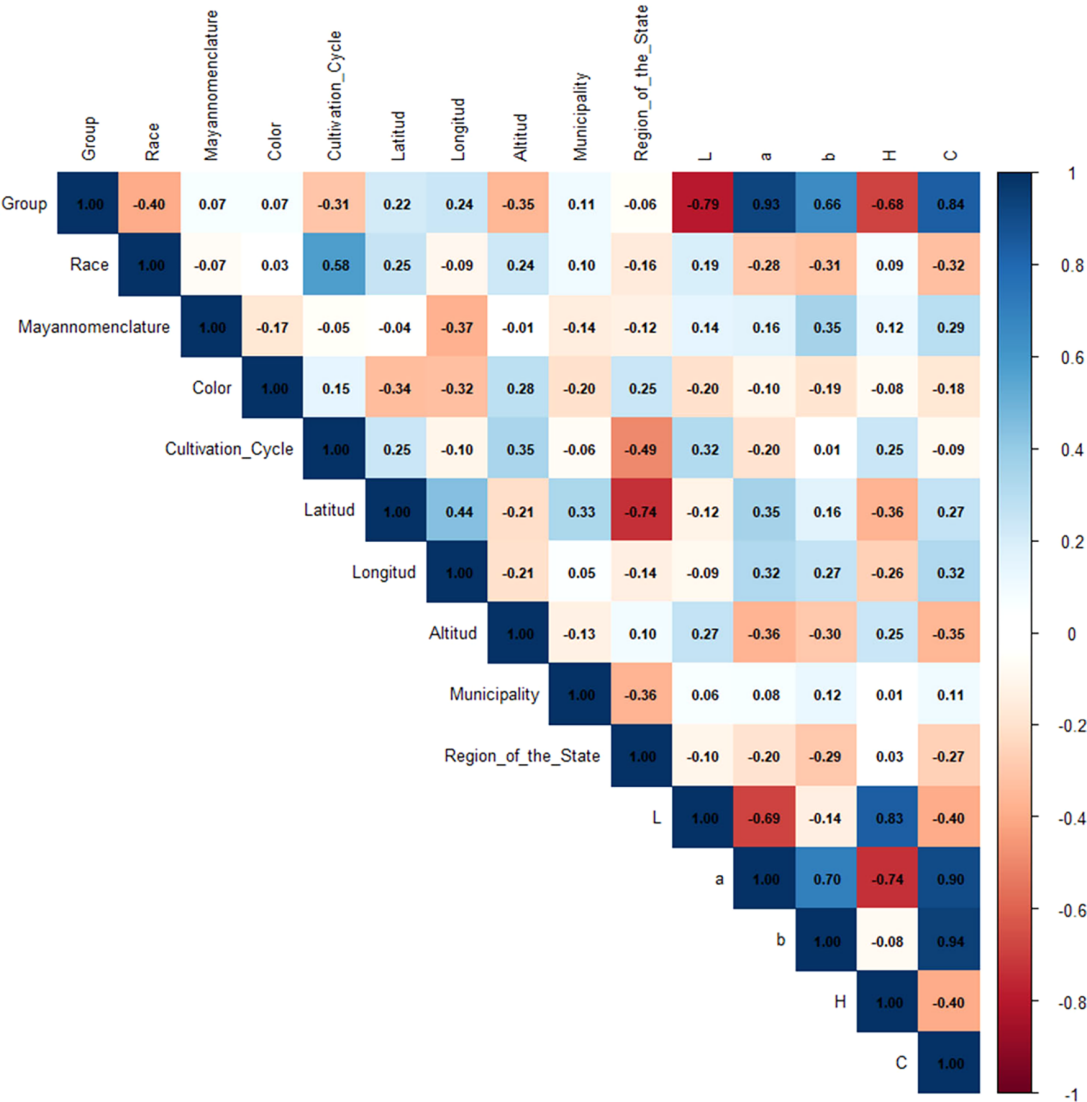
Correlation matrix metadata.

Following the clear separation observed between red and purple varieties through untargeted analysis (PCA), a univariate analysis was applied to the 512 detected molecular entities, reducing the number of significant compounds to 101 after the Benjamini-Hochberg correction. Ten unique compounds were identified in purple samples, three in red samples, and 35 shared between both populations (Figure 5A).

**Figure 5:**
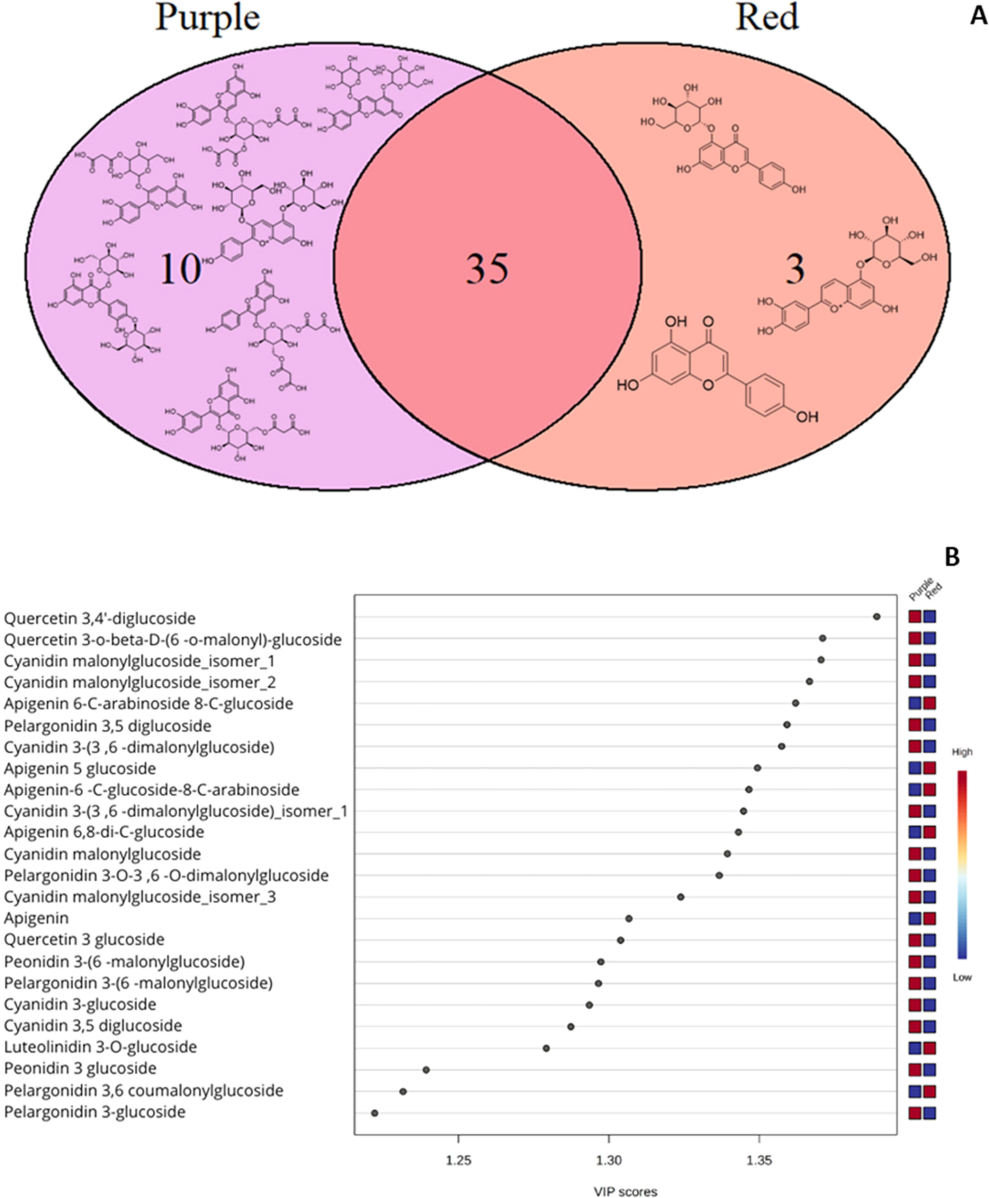
(A) Venn diagram of unique distinguishable compounds identified by color group (Purple and Red). (B) Projection Importance Values (VIP > 1) of key compounds that discriminate between red and purple maize, the identified compounds passed the Shapiro-Wilk tests and the Benjamini-Hochberg correction. After this, the isotopic profile was compared with the HMDB, FooDB and PubChem databases.

Principal component analysis (PCA) of the targeted set revealed a clear clustering between red and purple samples, with the first two principal components explaining 77.9% of the total variance (Supporting Figure S4). Likewise, partial least-squares-discriminant analysis (PLS-DA) showed that Projection Importance Values (VIP > 1), the most important compounds for discrimination were quercetin-3,4-diglucoside, quercetin-3-*O*-β-d-(6-*O*-malonyl)-glucoside, and isomers of cyanidin malonylglucoside in purple maize, while red maize was characterized by apigenin-6-C-arabinoside 8-C-glucoside and apigenin 5-glucoside (Figure 5B). This differential distribution of flavonoids suggests specific biosynthetic pathways associated with each variety’s pigmentation.^23^

Correlation analysis between compounds and metadata variables revealed distinct patterns (Figure 6). In the case of the growth cycle, distinctive patterns were observed in polyamine distribution. Early cycle varieties (<70 days) showed a strong positive correlation with spermidine (*r* = 0.82) and derivatives of pelargonidin and quercetin, while spermine exhibited a negative correlation (*r* = −0.75). This pattern suggests that early maturing plants prioritize compounds that favor rapid growth, where spermidine acts as a regulator of accelerated cell cycles.^24^ This spermidine accumulation could reflect a biochemical adaptation to complete development in a shorter time, possibly in response to abiotic stress conditions such as shallow soils, low water retention, or poor fertility.^25^ These conditions are frequently found in southern Yucatán, particularly in municipalities like Tixmehuac, Peto, and Tahdziú, where rocky Leptosol and Regosol soils limit water availability.^26^ In contrast, late-cycle varieties (>90 days) were dominated by spermine (*r* = 0.89), associated with long-term stress protection mechanisms, while showing low or no correlation with spermidine, quercetin, apigenin, and anthocyanin derivatives. This profile suggests that spermine may be linked to greater long-term cell growth and differentiation capacity, consistent with prolonged vegetative development, possibly favored by deeper, organic-rich soils like those found in central regions (e.g., Yaxcabá, Sotuta).^27^

**Figure 6:**
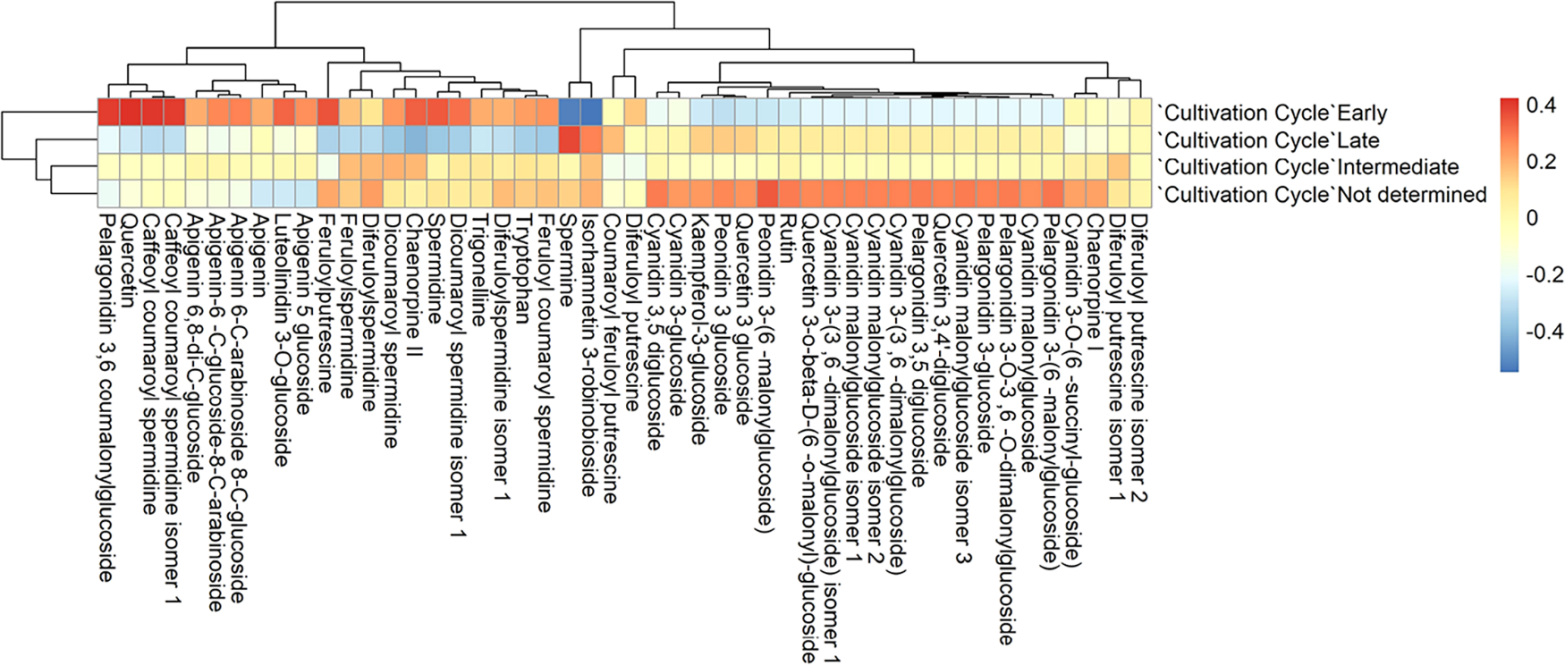
Maize cultivation cycle as a categorical variable studied. The early crop cycle corresponds to fewer than 70 days to flowering, the intermediate cycle ranges from 70 to 90 days, and the late cycle exceeds 90 days to flowering; correlations were calculated using Pearson’s coefficient, using the transformed values of each metabolite (log + Pareto scaling) and binary coding for categorical variables.

Midcycle varieties showed no strong correlations with any metabolites except a weak link to isorhamnetin derivatives.^28^ This lack of defined profiles could reflect genetic or phenotypic heterogeneity or intermediate ecological pressures.^29^ Taken together, these differences likely reflect divergent physiological strategies: early maturing varieties prioritize metabolic efficiency to complete their developmental cycle under limiting conditions, while late-maturing varieties allocate resources toward compounds that enhance resilience and maintain viability during prolonged stress.^30^ Therefore, it is suggested that early cycle maize does not exhibit detectable spermine concentrations. Spermidine and spermine concentrations both result from and influence the maize growth cycle. It has been shown that concentrations change during different growth stages, such as increasing dramatically after germination, and they also play a role in regulating growth processes and stress tolerance. For example, exogenous spermidine has been shown to improve seed germination and vigor in maize.^31^

Geographic distribution revealed metabolic patterns linked to specific edaphoclimatic conditions (Supporting Figure S5). In southern Yucatán (Tahdziú, Peto, Tixmehuac), characterized by acidic soils (pH 4.5–5.8) and low altitude (<100 masl), spermidine, tryptophan, and quercetin predominated, with a notable absence of spermine and anthocyanins. This profile suggests adaptation to nutrient-leached soils, where spermidine acts as an osmoprotectant and tryptophan compensates for nutritional limitations. In contrast, the central region (Sotuta, Uayma), with calcareous soils (pH 7.2–8.4) and higher altitude (250–400 masl), showed a predominance of spermine (*r* = 0.85) and absence of spermidine, indicating adaptation to intermittent water stress. The eastern zone displayed a unique profile dominated by apigenin and lacking cyanidin, possibly as an adaptive response to high UV radiation.^32^

Collectively, the results suggest that the distribution and relative abundance of polyamines like spermidine and spermine could serve as biochemical indicators of growth cycle and agroecological adaptation in maize varieties. Furthermore, it is hypothesized that the presence or absence of these polyamines is not merely a consequence of the growth cycle but an active determinant of it, depending on edaphic and climatic conditions. This relationship should be explored in complementary functional studies.

Analysis by race (Figure 7) revealed that Tuxpeo, associated with humid lowlands, correlated with cyanidin and quercetin derivatives but showed no link to apigenin. In contrast, Dzit Bacal and Nal Tel races, typical of the semiarid east, exhibited moderate correlations with apigenin. The presence of spermine in late Tuxpeño (*r* = 0.78) suggests artificial selection for resilience, while its absence in Nal Tel × Tuxpeño indicates incomplete hybridization of metabolic pathways. These patterns reflect how natural and artificial selection have shaped differentiated metabolic profiles based on cultivation conditions.^33^

**Figure 7:**
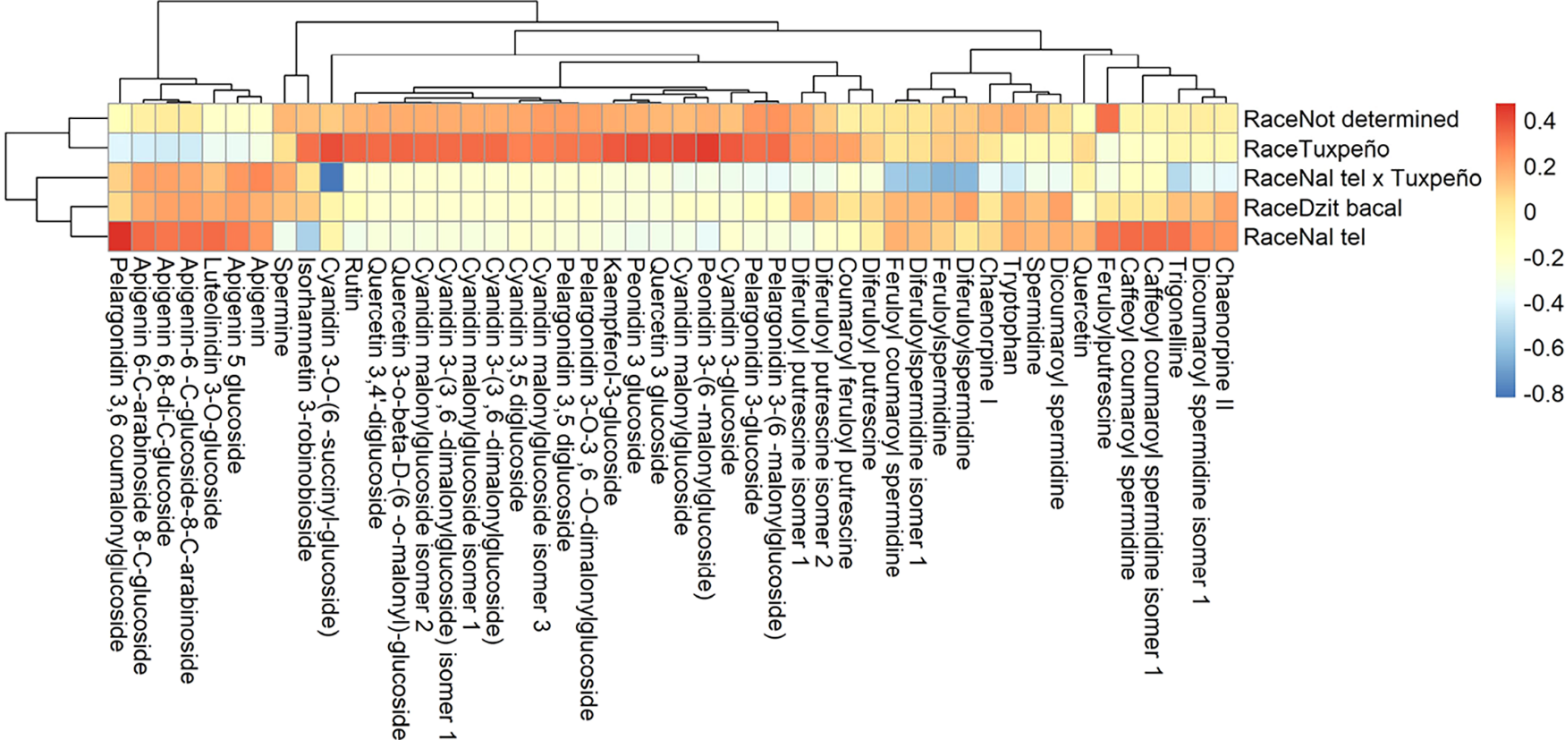
Correlation heatmap for normalized abundance of identified compounds against the maize race was studied as a categorical variable. Correlations were calculated using Pearson’s coefficient, using the transformed values of each metabolite (log + Pareto scaling) and binary coding for categorical variables.

Overall, the colorimetric parameters (CIE L*a*b) are reliable indicators of metabolic profiles in pigmented maize, with clear correlations between reddish tones (a) and apigenin, as well as darkening (L*) and anthocyanins.^34^ Metabolomic analyses confirmed distinct chemical profiles between red and purple varieties, with specific flavonoids (quercetin and cyanidin in purple; apigenin in red) suggesting differential biosynthetic pathways.^35^ Additionally, the distribution of polyamines like spermidine and spermine reflects agroclimatic adaptations, linking to growth cycles (early vs late) and regional edaphic conditions (acidic southern soils vs calcareous central soils). These findings highlight the close relationship between metabolic profiles, agronomic traits, and cultivation environments, proposing polyamines as potential adaptation biomarkers.^36^ Likewise, variability among races (Tuxpeo, Dzit Bacal, Nal Tel) demonstrates how natural and human selection has shaped unique chemotypes, reinforcing the importance of conserving this biochemical diversity for future breeding programs and sustainable utilization. The findings presented here provide valuable insights that may support the sustainable utilization, conservation, and genetic enhancement of Yucatecan native maize while reinforcing its agronomic, nutritional, and cultural importance. Furthermore, the metabolomic profiling highlights pronounced chemical diversity among varieties, indicating that variations in polyphenol, polyamine, phenolic amide, and alkaloid composition may play a key role in defining the unique sensorial and functional properties of each maize population.

## Supplementary Material



## Data Availability

The data presented in this study are available in the article.

## Notes

Sample collection map; chromatogram; spectra and additional PCA and heatmaps for the metabolomic profiling (PDF)
