# Supplementary material for: Metabolomic Profiling of Native Yucatan Maizes (Zea mays L.): A Strategy for Harnessing Biodiversity
Source: J Agric Food Chem. 2026 Jan 6;74(2):2405–17. doi: 10.1021/acs.jafc.5c09711 (PMC12833867; doi:10.1021/acs.jafc.5c09711)
Supplement: Supplementary file 1 [file jf5c09711_si_001.pdf]

Supporting Information

Metabolomic profiling of native Yucatan Maizes (*Zea mays* L.): A Strategy for Harnessing Biodiversity.

Elia María Ku Pech[a,b] Emilio Piña Betancourt[a,c], Claudia Balderas[a,d], Javier Orlando Mijangos-Cortés[b], Enrique Sauri Duch[c] and Sonia de Pascual-Teresa[a*].

[a] Departamento de Metabolismo y Nutrición, Instituto de Ciencia y Tecnología de Alimentos y Nutrición (ICTAN), Consejo Superior de Investigaciones Científicas (CSIC), Jose Antonio Novais 6, Madrid 28040, Spain.

[b] Centro de Investigación Científica de Yucatán, Chuburná de Hidalgo, 97205 Mérida, Yucatán, México.

[c] Instituto Tecnológico de Mérida, Tecnológico Nacional de México, 97118 Mérida, Yucatán, México.

[d] Instituto de Química Orgánica General (IQOG), Consejo Superior de Investigaciones Científicas (CSIC), Juan de la Cierva 3, Madrid 28006, Spain.

*Corresponding authors: s.depascualteresa@csic.es

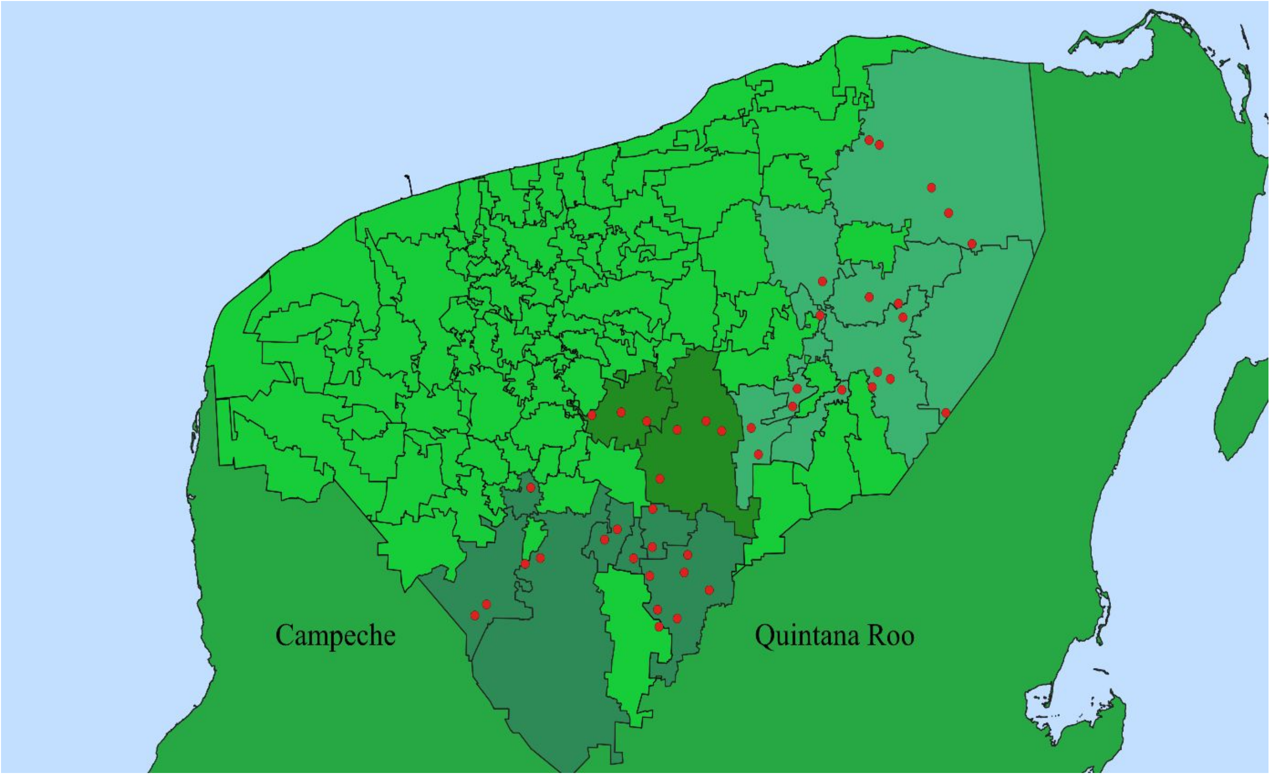

Supplementary Figure 1. Collection map of the studied accessions. The map corresponds to the Yucatan Peninsula; the red dots indicate the areas sampled for the collections established in **Table 1**.

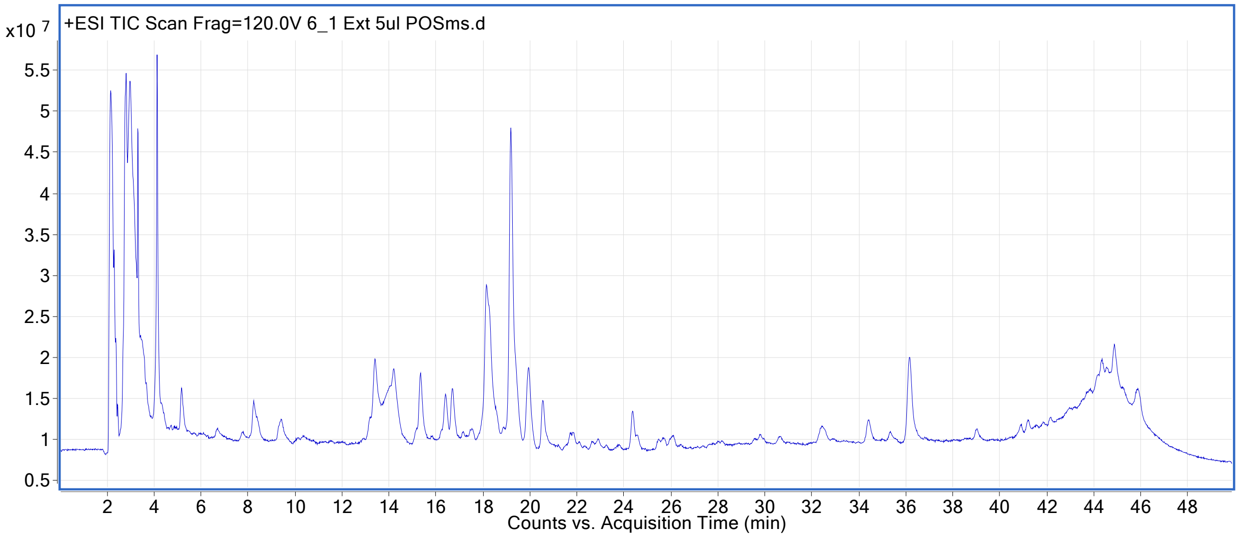

Supplementary Figure 2. Total ion chromatogram (TIC) for sample 6 (R) under the chromatographic and MS conditions described in the methodological section.

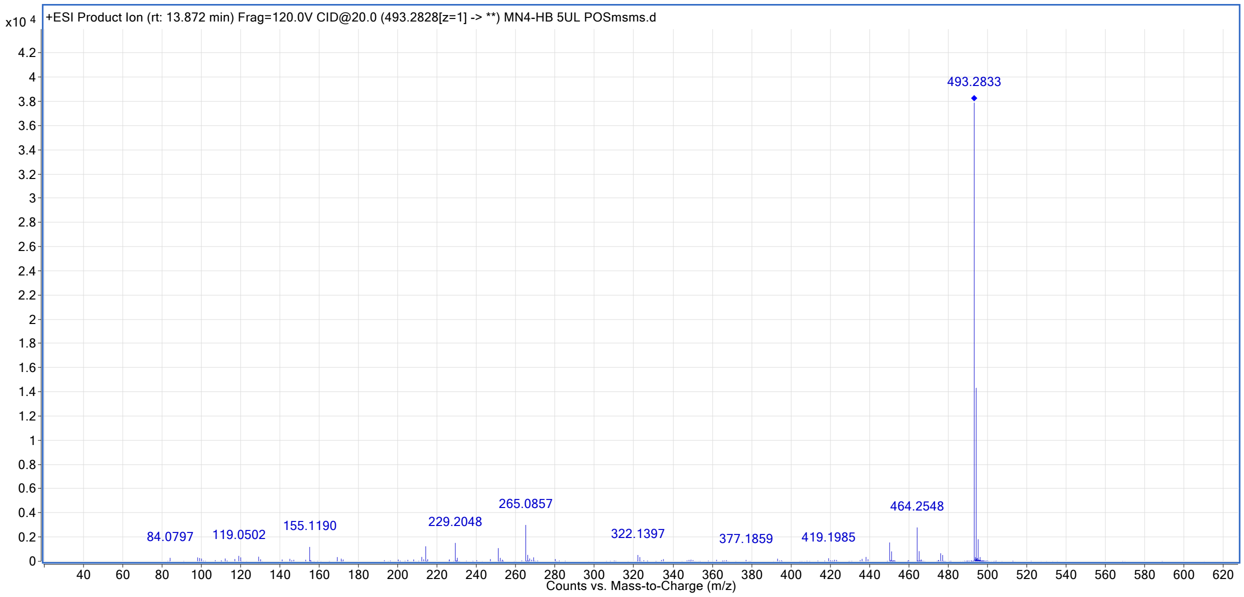

Supplementary Figure 3. MS/MS fragmentation analysis of m/z 493.2806 ($[M+H]^+$) identified as being chaenorpine in agreement with Zhang & Peterson (2018), with molecular formula C28H36N4O4 and with a major fragment at 265, and in our case 155.

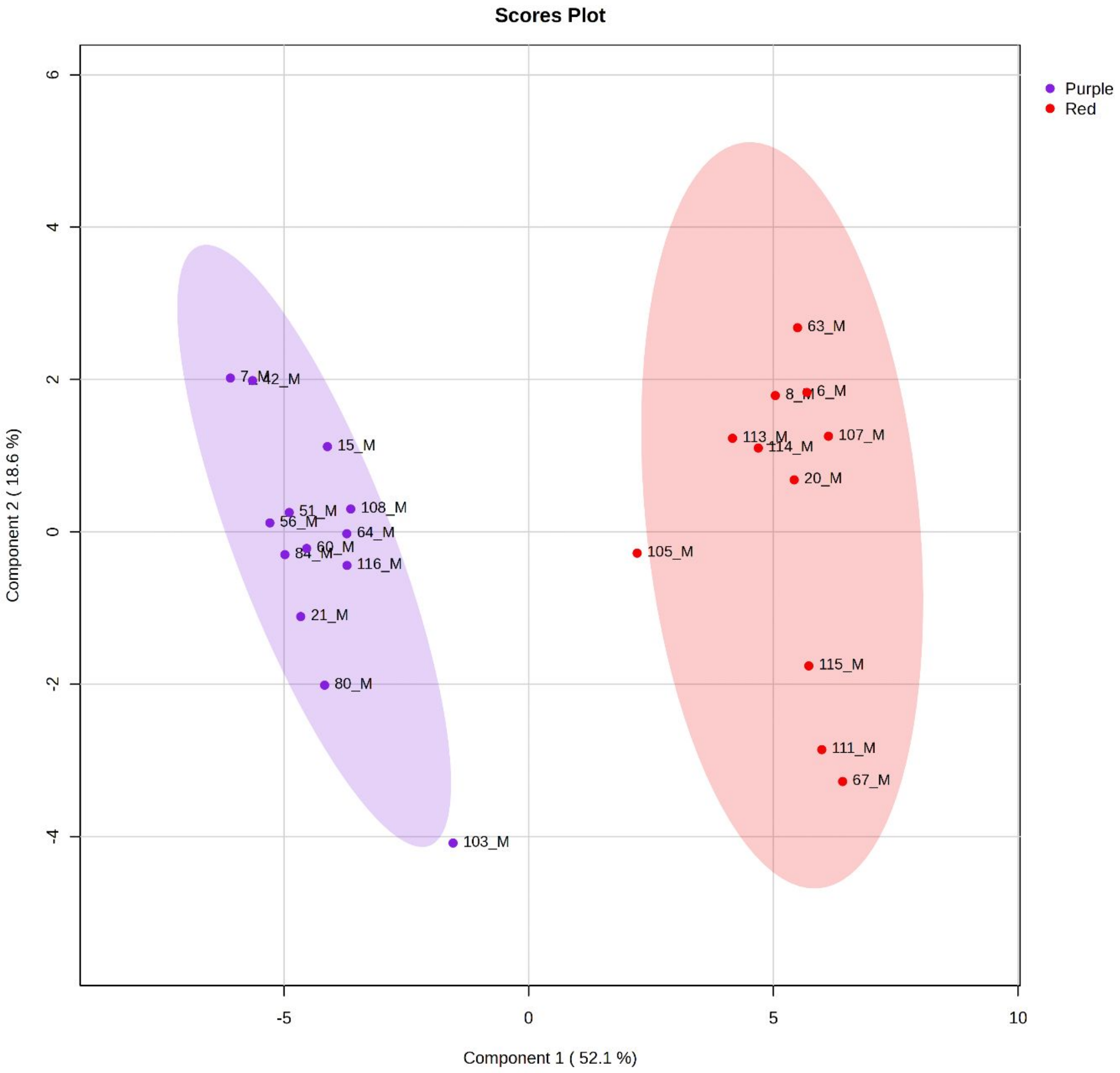

Supplementary Figure 4. PCA of Targeted Metabolites Showing Distinct Clustering of Red vs. Purple Samples (PC1+PC2 = 70.7% Variance).

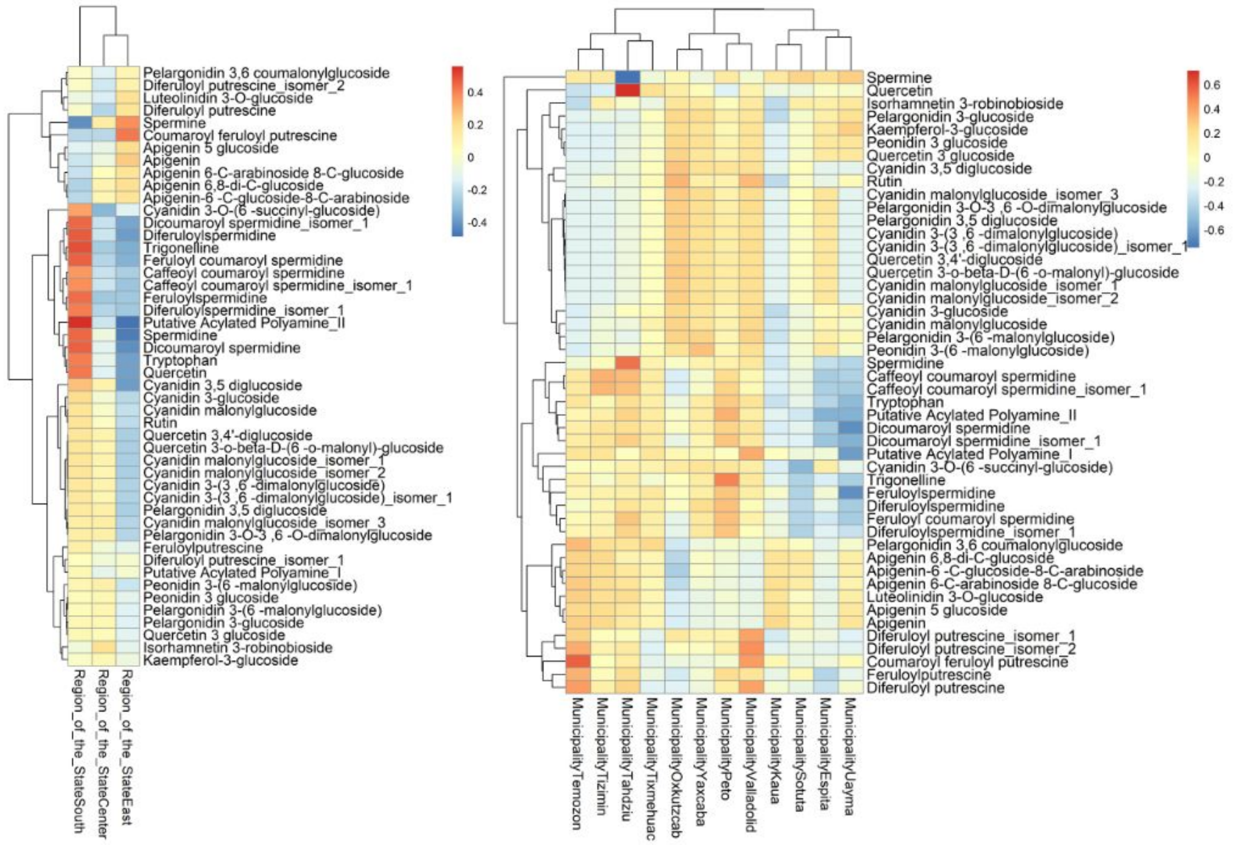

Supplementary Figure 5. Correlation Heatmap of Identified Metabolites Across Yucatán Regions (South, Center, East) and Municipalities, Highlighting Edaphoclimatic Associations.
